# Association between Weight for Length and the Severity of Respiratory Morbidity in Preterm Infants

**DOI:** 10.3390/children11010091

**Published:** 2024-01-12

**Authors:** Pradeep Alur, Kristen Harvey, Kyle Hart, Wondwosen K. Yimer, Renjithkumar Kalikkot Thekkeveedu

**Affiliations:** 1 Hampden Medical Center, Penn State Health, Harrisburg, PA 17025, USA; 2 School of Medicine, University of Mississippi Medical Center, Jackson, MS 39216, USA; 3 Department of Data Sciences, University of Mississippi Medical Center, Jackson, MS 39216, USA; 4 Department of Pediatrics, Deaconess Women’s Hospital, Newburgh, IN 47630, USA

**Keywords:** preterm, weight, length, BMI, BPD

## Abstract

Association Between Weight for Length and the Severity of Respiratory Morbidity in Preterm Infants. Objective: To determine whether higher weight-to-length z-scores after 32 weeks of gestation are associated with higher pulmonary scores (PSs) in preterm infants requiring respiratory support using a prospective observational study. Methods: Infants born at <30 weeks, with a post-menstrual age (PMA) of 30–33 weeks, were enrolled. The infant’s weight, length, and head circumference were measured weekly. Data on calories/kg/d, protein g/kg/d, weight-for-length percentiles, z-scores, and BMI at 33 through 40 weeks PMA were collected. The PS was calculated. Results: We analyzed 91 infants. The mean gestational age was 26.9 ± 1.7 weeks. The mean birthweight was 0.898 ± 0.238 kgs. They were predominantly African American (81.3%) and girls (56%). Postnatal steroids were administered in 26.4% of the infants. The mean duration of invasive ventilation was 19.23 days ± 28.30 days. There was a significant association between the PS and W/L z-score (*p* < 0.0001). For every one-unit increase in W/L z-score, the PS increased by 0.063. There was a significant association between the PS and W/L percentile (*p* = 0.0017), as well as BMI (*p* ≤ 0.0001). For every unit increase in W/L percentile, the PS increased by 0.002, and for a unit increase in BMI, the PS increased by 0.04. The association remained significant after postnatal steroid use, sex, and corrected and birth gestational ages were included in the regression analysis. Nutrition did not affect the anthropometric measurements. Conclusions: Our study is the first to demonstrate that a higher BMI and W/L may adversely affect the respiratory severity in preterm infants. Studies with larger sample sizes are needed to confirm our findings.

## 1. Introduction

Extremely low-birthweight premature infants are at high risk of bronchopulmonary dysplasia (BPD) [1,2], which is a significant contributor to chronic pulmonary morbidity in preterm infants. The incidence of BPD decreases as the gestational age at birth increases. Severely affected lungs may not be able to keep up with the increased metabolic demands of the body. This may be further complicated if the body weight outgrows the linear growth, as severely affected lungs may not be able to keep up with the increased metabolic demands of the body.

The BPD collaborative suggests [3] that the weight for length should be maintained at the 50th percentile. Hence, it is a common practice among clinicians to restrict calories when the weight-for-length ratio is high. In a recent meta-analysis that looked into the outcomes in mechanically ventilated adults and their association with BMI, it was noted that the duration of mechanical ventilation and length of stay in an intensive care unit was longer in patients with a BMI of 30–39.9 kg/m^2^ than in those with a BMI of 18.5–24.9 kg/m^2^ patients [4]. Adiposity has also been shown to affect lung function in children and adolescents negatively. A higher BMI in children is also considered an independent risk factor for wheezing and enhanced medication use [5,6]. Research has also found that children with a higher adiposity have a lower FEV1/FVC ratio [7,8]. The effects of higher adiposity on altered lung functions may also be true in young children. Miller et al. found that infants whose growth in length was deteriorating were more likely to require increased oxygen during a respiratory wean and were at a higher risk of developing pulmonary hypertension [9]. However, no studies indicate whether a higher weight for length is detrimental in preterm infants with respiratory morbidity. It is also unknown whether a higher BMI or higher weight-for-length ratios indicate excessive adiposity in preterm infants.

The large multinational Intergrowth 21st study has published normative values for the weight for length in preterm infants. The study concluded that weight for length is a more accurate predictor of fat-free mass (FFM) and fat mass (FM) than body mass index (BMI) in premature infants, as measured using air displacement plethysmography in premature infants [10]. Thus, the weight for length in preterm infants can be a helpful non-invasive tool to assess the perturbations in body composition. However, there is no current evidence associating anthropometric measurements in preterm infants with BPD and their respiratory outcomes.

DEXA (dual-energy X-ray absorptiometry) and air displacement plethysmography accurately estimated the fat mass and fat-free mass in preterm and term infants. However, these methods are impractical in sick neonates, and air displacement plethysmography cannot be used on infants requiring supplemental oxygen. Several experts have recommend assessing weight status by monitoring the weight for length in children < 2 years of age [11,12].

This study investigated the association between the weight-for-length (W/L) percentiles and respiratory morbidity in preterm infants. We therefore explored our hypothesis that higher percentiles and z-scores for weight for length in preterm infants could increase the severity of pulmonary morbidity. We used the pulmonary score (PS) to assess the severity of pulmonary morbidity in preterm infants. Our goal was to determine whether higher weight-to-length ratios after 32 weeks of gestation in preterm infants born at less than 30 weeks of gestation (at high risk for bronchopulmonary dysplasia) are associated with a higher PS before term-corrected gestational age, requiring respiratory support, in a prospective observational study.

## 2. Methods

This project was supported by the Pilot Project Grant Program of the Mississippi Center for Clinical and Translational Research (MCCTR) and the NIH: Award #1U54GM115428. The University of Mississippi Medical Center’s institutional review board approved this prospective study in February 2019, and the approval number is IRB File #2018-0204.

Inclusion: We enrolled preterm infants born at less than 30 weeks of gestation, with a post-menstrual age (PMA) of 30–33 weeks, and requiring oxygen at enrollment for at least three days at the time of enrollment.

Exclusion: We excluded babies with congenital anomalies such as abdominal wall defects, skeletal dysplasia affecting the chest wall or lungs, congenital diaphragmatic hernia, babies with myopathies, confirmed surfactant protein-B deficiency, or congenital prostaglandin-dependent heart lesions from the study.

The institutional review board at the University of Mississippi Medical Center approved the study, and informed consent was obtained from each participant. The study was performed as per the Declaration of Helsinki.

### 2.1. Data Collection

A dedicated research nurse was trained to obtain accurate anthropometric measurements based on the American Academy of Pediatrics’ recommendations for anthropometric measurement standard operating procedures (https://www.aap.org/en/patient-care/newborn-and-infant-nutrition/newborn-and-infant-nutrition-assessment-tools/anthropometric-measurements/, accessed on 2 January 2024). The research nurse accurately measured the weight (in grams) using a certified electronic weighing scale, the length using an infant length board (in centimeters), and the head circumference (in centimeters) using a paper-based measuring tape once a week of all the recruited infants. Each parameter was measured twice for accuracy and we calculated a mean for each parameter. The weight-for-length percentiles and z-scores for the corresponding week were obtained using the Intergrowth 21st study data. We collected data on calories/kilogram/day, protein grams/kilogram/day, protein-to-calorie ratio, weight-for-length percentiles, z-scores, and BMI from 33 through 40 weeks PMA. The definition of BPD used in the current study was as per the NICHD BPD workshop published in February 2018 [13].

### 2.2. Pulmonary Score (PS)

The PS is a standardized score that assesses the severity of pulmonary morbidity in premature infants with bronchopulmonary dysplasia (BPD), developed by Ashima Madan et al. for the STOP-ROP study [14]. The PS scores for the current study were obtained daily, and the mean pulmonary scores were computed weekly from 33 weeks until 40 weeks of PMA or discharge, whichever occurred early, for stratification and subsequent comparison purposes The score for a patient’s respiratory support level can be calculated using the following formula: (Fio2) (support) + (medications). In this formula, Fio2 is expressed as a fraction of the room air (0.21) for a ventilator, CPAP, or hood but as an effective Fio2 for a nasal cannula. The support score is 2.5 for a ventilator or tracheostomy, 1.5 for CPAP (nasal or endotracheal), and 1.0 for a nasal cannula, hood oxygen, or no oxygen. The medication score is 0.20 for systemic steroids for chronic lung disease, 0.10 for regular diuretics (daily or every other day) or inhaled steroids, and 0.05 for methylxanthines or intermittent diuretics. The normal PS ranges from 0.21 to a maximum of 2.7.

### 2.3. Statistical Analysis

We compared the mean weight-for-length percentiles and z-scores with the PS.

### 2.4. Sample Size Calculation

No previous studies have compared PS scores with weight-for-length percentiles. Therefore, to calculate the sample size, we assumed that the mean PS score would be 0.59 ± 0.22 in the high weight-for-length group vs. 0.44 ± 0.14 in the normal weight-for-length group. This assumption was based on a study that used the PS to differentiate infants with BPD who passed the room air challenge from those who failed to wean to room air from a nasal cannula [15]. For the study, we classified the weight-for-length percentile as high if it was 50 or higher and normal if the weight-for-length percentile was <50. Based on our assumptions of an 80% probability of rejecting the null hypothesis (OR = 1) when it was false, a 5% probability of obtaining a false positive with the (two-sided) statistical test, and the mean PS in the high weight-for-length group being 0.59 ± 0.22 vs. 0.44 ± 0.14 in the normal weight-for-length group, we would need a sample size of 46 in each group.

The data were analyzed using both descriptive and inferential statistical techniques. Summary statistics were computed as the mean (standard deviation) for continuous variables and frequency (percentage) for categorical variables. A linear mixed model that accounted for the correlation due to repeated measurements for each baby was employed to assess the association between the response and independent variables of interest, adjusting for covariates. The statistical significance was assessed as a *p*-value < 0.05 based on two-tailed tests.

## 3. Results

We analyzed 91 infants in our study. We excluded one infant after he was too sick to obtain further anthropometric measurements. The mean gestational age was 26.9 ± 1.7 weeks (range—23 weeks to 29.9 weeks). The mean birthweight was 0.898 ± 0.238 kgs = (range—0.42 kg to 1.52 kg). The babies were predominantly African American (81.3%) and girls (56%). Postnatal steroids were administered in 26.4% of the infants. The mean duration of invasive ventilation was 19.23 days ± 28.30 days. The mean duration of non-invasive ventilation was 44.03 ± 22.60 days. The mean PS at 36 PMA was 0.36 ± 0.41. Table 1 shows the maternal and neonatal characteristics.

### 3.1. Anthropometric Measures and Post-Menstrual Age

**Figure 1:**
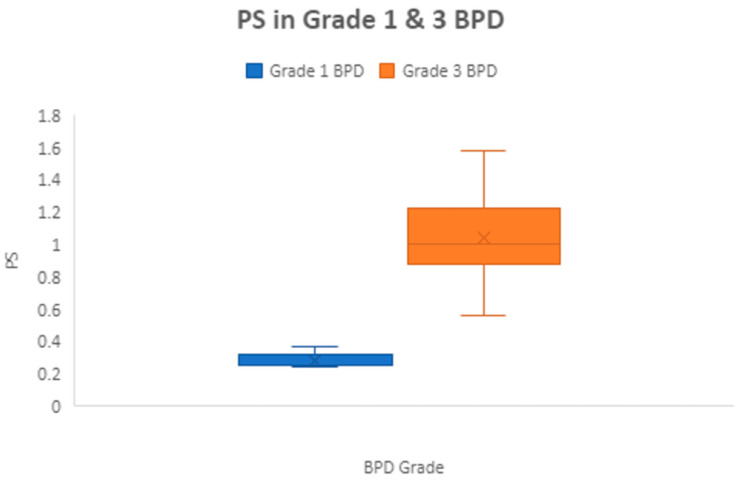
Correlation between BPD severity and pulmonary severity scores.

The mean PS was significantly higher in grade 3 BPD (1.04 ± 0.4) compared to grade 1 BPD (0.29 ± 0.05), *p* ≤ 0.0001 (Figure 1), showing that the PS correlated with pulmonary morbidity. Between 33 to 40 weeks PMA, 113 infant measurements had W/L measurements above the 50th percentile. Of these 113 measurements, 92 (82.4%) had a normal length for PMA. This suggests that high weight/length is due to a higher weight for age and not due to growth restriction in length in most cases. Similarly, at 36 PMA, 84.6% of those with a W/L percentile > 50 also had normal length for PMA.

### 3.2. Correlation between Anthropometric Measures and Pulmonary Severity (PS)

**Figure 2:**
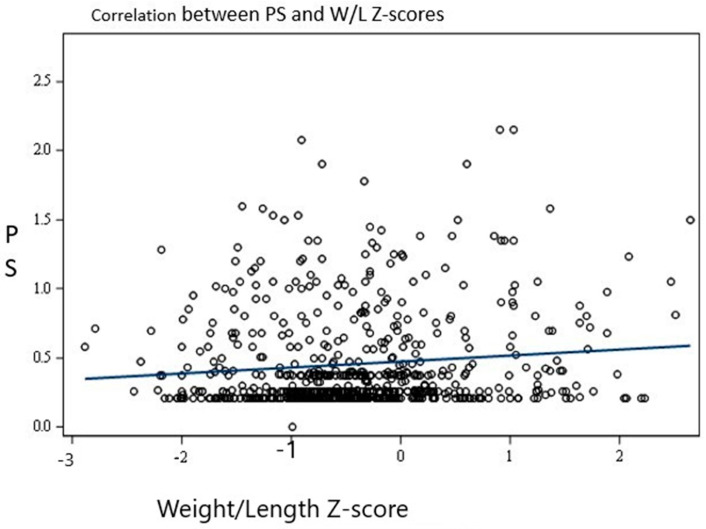
Association between pulmonary score and W/L z-scores.

Overall, there was a significant positive association between the PSs and W/L z-scores (*p* < 0.0001). For every one-unit increase in the W/L z-score, the PS increased by 0.063. This is illustrated in Figure 2.

Similarly, there was a significant positive association between the PSs and W/L percentiles (*p* = 0.0017) as well as BMI (*p* ≤ 0.0001). For every one-unit increase in the W/L percentile, the PS increased by 0.002, and for a unit increase in BMI, the PS increased by 0.04.

### 3.3. Effect of Regression Analysis

The association between the PSs and W/L z-scores, W/L percentiles, and BMI remained significant (*p* ≤ 0.0001) after postnatal steroid use, sex, and post-menstrual and birth gestational ages were included in the regression analysis, which suggests that the association between the PS and anthropometric measurements is natural.

### 3.4. Effect of Postnatal Steroids

Postnatal steroids were used in 26.4% of infants. While 83.3% of infants with grade 3 BPD received postnatal steroids, only 30% of those with grade 1 BPD received postnatal steroids. Since postnatal steroids may affect growth in general, we analyzed the results after excluding the infants exposed to postnatal steroids to evaluate whether the W/L and PS association was true. The association between the PS and W/L z-score remained significant after the infants receiving postnatal steroids were excluded from the regression analysis. After excluding infants exposed to postnatal steroids, for every unit increase in the W/L z-score, the PS increased by 0.03 with a *p*-value of 0.003.

### 3.5. Effect of Nutrition and Sex

There were no sex differences observed in the mean PS. The mean PS was 0.65 ± 0.32 in the male infants and 0.66 ± 0.49 in the female infants. (*p* > 0.1). The incidence of BPD was 28% among females compared to 45.9% in males (*p* = 0.28). The incidence of BPD was 29.4% among white infants compared to 37.1% among African American infants and was not statistically significant with *p* = 0.55. Likewise, no association was noted between the anthropometric measurements and the nutrition provided to the infants in the study. The mean calories provided at 36 weeks PMA was 121.39 ± 18.28 Kcal/kg/d. The mean protein provided at 36 weeks PMA was 3.78 ± 0.59 g/kg/d. The mean calories did not correlate with BMI (*p* = 0.78). Similarly, mean protein did not correlate with BMI (*p* = 0.83). Likewise, mean calories or mean protein did not correlate with the W/L percentiles (*p* = 0.07 and 0.48, respectively), indicating that nutrition did not play a role in higher W/L or BMI in the current study.

### 3.6. Predicting ROC (Receiver Operator Characteristic) Curves with Pulmonary Scores

We used pulmonary severity scores as a predictor variable and weight-for-length z-score as a response variable to solve a binary classification problem. We also assumed various W/L z-scores such as 0.4 and percentiles of 40%, 50%, 75%, and 90% to determine the best ROC curve. However, a W/L z-score of 0.4 and a W/L percentile of 75 had the highest AUC (area under the curve). A weight-for-length z-score of 0.4 or higher indicates abnormal growth, while lower values indicate typical growth.

To evaluate the performance of our classifier at different thresholds, we used a receiver operating characteristic curve. The ROC curve plots the true positive rate (TPR) against the false positive rate (FPR) for each threshold. The TPR is the proportion of cases with abnormal growth correctly classified and the FPR is the proportion of cases with average growth incorrectly classified.

The AUC (area under the curve) is the integral of the ROC curve, and it measures the overall accuracy of the classifier, regardless of the threshold. The AUC ranges from 0 to 1, with 0 meaning the classifier is entirely wrong and 1 meaning it is correct.

**Figure 3:**
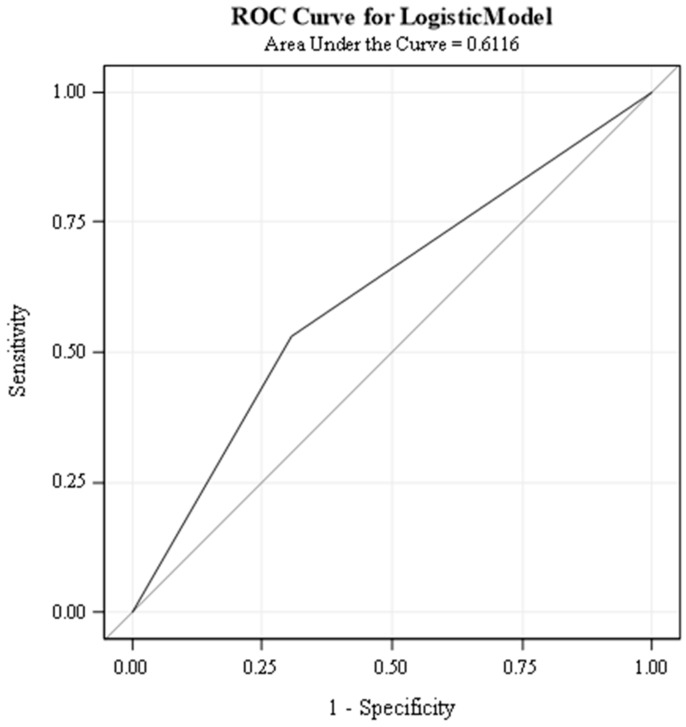
ROC curve for PS prediction with W/L Z-score of 0.4.

Our classifier attained an AUC of 0.6116 for a weight-for-length z-score of 0.4 (Figure 3). This means pulmonary scores can correctly identify the true class of 61.16% of cases. An AUC of 0.6116 suggests some predictive ability, but it is not considered a very strong result. Similarly, the W/L percentile of 75% had an AUC of 61.64%. While this is moderately better than random guessing, we feel a larger sample size is probably required for predicting a robust AUC.

## 4. Discussion

Our prospective observational study investigated the relationship between pulmonary scores (PS) and anthropometric indicators, such as weight-for-length (W/L) z-scores, W/L percentiles, and body mass index (BMI), in preterm infants. We found a significant positive correlation between the PS and these indicators. Specifically, the PS was found to be higher in grade 3 BPD compared to grade 1 BPD, similar to another study [16]. Our study’s findings suggest that as the W/L z-scores or percentiles increased, the PS worsened, indicating an increased risk of pulmonary complications in preterm infants with higher W/L ratios. This information can help identify those infants who are at a higher risk of developing pulmonary complications and in developing interventions to reduce the risk of such complications.

We investigated whether specific weight-for-length (W/L) thresholds could identify children with significantly higher pulmonary severity scores. We explored various W/L percentiles and z-score points. Remarkably, only two thresholds emerged as strong predictors: a W/L z-score of 0.4 and a W/L percentile of 75. Both thresholds yielded high areas under the ROC curve (AUCs), 61.16% and 61.64%, respectively, indicating a good discriminative ability. However, the relatively small sample size suggests some cautiousness in interpreting these cut-off values. A larger study could strengthen the evidence and more precisely define a reliable W/L threshold for identifying children at risk of higher pulmonary severity.

It is widely accepted that improved growth in very low-birthweight (VLBW) infants with bronchopulmonary dysplasia (BPD) is linked to better lung function [17,18]. However, there is little research on how abnormal growth affects lung function in premature babies. This paper is the first prospective study that focused on the association between altered body proportionality and the severity of respiratory status. A recent study looked into whether changes in the BMI of preterm infants from birth to 36 weeks of post-menstrual age are associated with the likelihood of developing BPD [19]. The study found that increases in the BMI z-score from birth to 36 weeks PMA were linked to higher odds of BPD and an increase in its severity. After adjusting for other factors, the study found that higher quartiles of the ΔBMI z-score were associated with higher odds of BPD. Specifically, the odds of developing BPD were significantly higher in quartile 3 (Q3) compared to quartile 2 (Q2), and in quartile 4 (Q4) compared to Q2. Our study associated pulmonary severity with increasing BMI and W/L z-scores and percentiles from 33 weeks PMA. Our study has revealed that preterm infants with a higher body mass index (BMI) or a larger weight-to-length ratio are at a greater risk of developing respiratory health issues, even before they are diagnosed with bronchopulmonary dysplasia (BPD). These respiratory issues can lead to serious complications that can affect infants’ overall health and development [3], although we did not specifically evaluate the impact of BPD on co-morbidities. It is, however, important to closely monitor and maintain the appropriate anthropometry in preterm infants who are at risk of developing BPD. Proper nutrition, feeding practices, and weight management can help reduce the risk of respiratory illness and improve the long-term health outcomes in preterm infants.

The results of our study indicate that all three anthropometric measurements, namely the W/L percentiles, W/L z-scores, and BMI, were found to be significantly associated with respiratory morbidity. These findings underline the importance of considering these anthropometric measures while assessing respiratory health. However, it is worth noting that our study was not designed to predict the development of BPD, and further research is needed in this area.

Recent studies have shown that administering postnatal steroids, either single or multiple courses, has no effect on the growth of premature infants with bronchopulmonary dysplasia (BPD) [20,21,22]. However, to ensure that the use of postnatal steroids did not influence the observed results, we analyzed the data after eliminating the infants exposed to postnatal steroids. In our study, approximately 26% of the infants had received postnatal steroids. We therefore conducted a regression analysis after removing all the infants who received postnatal steroids. The results showed that the relationship between the PS and weight/length z-score remained significant (with a point estimate of 0.03 and a *p*-value of 0.003). This confirms that there is a genuine link between PS and anthropometric measurements, adding strength to our findings.

Our study found no association between calorie and protein intake and BMI or weight/length (W/L) percentiles in infants with severe lung disease. This means there was no correlation between the infants’ calorie or protein intake and their BMI or W/L percentiles. Our findings do not support the hypothesis that excessive nutrition intake is responsible for a higher BMI or W/L percentile in our study’s infants. Therefore, we conclude that the association between PS and anthropometric measures is natural.

Our research has confirmed the belief among clinicians that infants with pulmonary morbidity may be adversely affected by a higher weight-to-length ratio or body mass index. The BPD collaborative suggests [3] that maintaining weight for length at the 50th percentile is beneficial, and our study supports the current recommendation. The strengths of our study are that it is prospective and includes preterm infants at increased risk of BPD. We have assessed pulmonary severity in relation to anthropometric data. A dedicated research nurse was responsible for measuring the anthropometric data, ensuring high consistency in the results.

Our study has a few limitations. We did not investigate whether improving the body mass index or weight-to-length ratio would reduce respiratory severity. A higher W/L percentile could be due to having a higher weight or a lower length for age, while the other variables remain normal. However, it is possible that the higher pulmonary morbidity in our study group could have stunted length growth, resulting in normal weight for age, lower length for age, and deceptively higher W/L percentiles. However, we noticed that most of those with weight-to-length percentiles greater than 50 at 36 weeks of PMA also had normal lengths for PMA. Hence, stunted length is not the cause of the higher W/L percentiles in the study group. This observation supports our hypothesis that a true association exists between higher weight-to-length percentiles and pulmonary severity.

Recent scientific research has demonstrated that optimizing the nutrition of premature infants between 31 and 34 weeks of corrected gestational age can have a positive impact on their body mass index (BMI) proportionality at the time of their discharge from hospital [23]. According to a recent study, providing the optimal nutrition to infants during their initial months is crucial for their healthy development. The study revealed that infants who received treatment as per the targeted nutrition guidelines had a significant reduction in disproportionately low BMIs (8.6% vs. 2.5%; *p* = 0.0380). This finding highlights the importance of following the proper nutrition guidelines during this early period to ensure the healthy growth and development of infants. A few studies have correlated the weight-for-length ratio with neonatal adiposity in term infants [24,25]. However, there are no similar studies in sick preterm infants. Our study’s findings add to the increasing body of evidence that emphasizes the need for more research on the impact of body proportionality indices on respiratory morbidity in preterm infants. It is crucial to comprehend the underlying mechanisms and create focused interventions to alleviate the burden of respiratory morbidity in this vulnerable population.

## 5. Conclusions

Our study is the first to demonstrate that a higher BMI and W/L may adversely affect the respiratory severity in preterm infants. Our findings suggest that maintaining the ideal anthropometry in preterm infants with severe respiratory conditions may prevent further respiratory morbidity in this subset of infants. Studies with larger sample sizes are needed to confirm our findings.

## Figures and Tables

**Table 1:** Maternal and newborn characteristics.

Maternal Characteristics	Number (Percentage)
Maternal diabetes	13 (15.5%)
Pre-eclampsia	41 (47.7%)
Antenatal steroid use	67 (74.4%)
Chorioamnionitis	11 (12.6%)
**Newborn characteristics**	
Male	40 (44%)
Black	74 (81.3%)
Methyl xanthine use	91 (100%)
Postnatal steroid use	24 (26.4%)
Pulmonary HTN	18 (19.7%)
Nitric oxide use	8 (8.8%)
Sildenafil use	1 (1.1%)
Diuretic use	30 (33%)
Any IVH	29 (31.9%)
Any ROP	22 (24.2%)
PDA present after 30 weeks	37 (40.6%)
PDA treatment	19 (20.9%)
Any BPD at 36 weeks	31 (34%)
Grade 3 BPD	18 (19.7%)
Ventilated babies at 36 weeks	14 (15.4%)
Ventilated babies at 40 weeks	5 (5.5%)
Culture-positive infection	24 (26.4%)
NEC	9 (9.9%)

## Data Availability

The data presented in this study are available on request from the corresponding author. The data are not publicly available due to the need for approval from the University of Mississippi Medical center IRB.
